# Is there an association between a plant-based eating pattern and clinical findings of a migraine headache?

**DOI:** 10.3389/fnut.2023.1117740

**Published:** 2023-07-04

**Authors:** Elham Karimi, Shahnaz Amani Tirani, Elham Sadat Azimi, Gholamreza Askari, Atefeh As’habi, Arman Arab

**Affiliations:** ^1^Department of Clinical Nutrition, School of Nutrition and Food Sciences, Isfahan University of Medical Sciences, Isfahan, Iran; ^2^Research Development Center, Arash Women’s Hospital, Tehran University of Medical Sciences, Tehran, Iran; ^3^Department of Community Nutrition, School of Nutrition and Food Sciences, Isfahan University of Medical Sciences, Isfahan, Iran; ^4^Isfahan Neurosciences Research Center, Alzahra Hospital, Isfahan University of Medical Sciences, Isfahan, Iran; ^5^Food Safety Research Center (salt), Semnan University of Medical Sciences, Semnan, Iran

**Keywords:** plant-based diet index, migraine, headache, diet, integrative medicine, Iran

## Abstract

**Aims:**

The present study was conducted among Iranian individuals diagnosed with migraine headaches to investigate the association between plant-based eating patterns and the clinical features of a migraine headache.

**Methods:**

In total, 262 patients aged 20–50 years who were diagnosed with migraine headaches were included in the current cross-sectional study. Participants’ dietary intake was assessed using a validated 168-item, semi-quantitative food frequency questionnaire (FFQ). Three versions of the plant-based diet index (PDI) comprising overall PDI, healthy PDI (hPDI), and unhealthy PDI (uPDI) were generated. Headache frequency, duration, and severity were assessed via a 30-day headache diary. Migraine-related disability was assessed with a validated version of the headache impact test (HIT-6) questionnaire. Serum nitric oxide (NO) was measured using the Griess method utilizing serum samples.

**Results:**

After controlling for potential confounders, an increase in mean PDI score from 48.22 to 61.67 decreased the frequency of headaches by −2.95 (95% CI: 0.06, 0.53; *p*_trend_ = 0.005). PDI was inversely associated with serum levels of NO in those with the highest scores of PDI compared to those with the lowest scores after adjustment for confounders (β = −7.69; 95% CI: −14.68, −0.69; *p*_trend_ = 0.028). After adjustment for potential confounders, an increase in the mean hPDI score from 47.96 to 65.25 decreased serum levels of NO by −11.92 (95% CI: −19.74, −4.10; *p*_trend_ = 0.003).

**Conclusion:**

Our findings suggest that higher adherence to a plant-based eating style, as evidenced by higher PDI and hPDI values, was significantly associated with lower headache frequency and serum levels of NO.

## Introduction

Migraine is a chronic neurologic disorder characterized by recurrent episodes of headache accompanied by nausea, vomiting, photophobia, and/or phonophobia (1). Migraine imposes substantial economic impacts on individuals and society resulting from loss of productivity and healthcare costs (2). Its global prevalence is 11.6%, which is higher among women than men with 13.8 and 6.9% prevalence, respectively (3). Moreover, the prevalence of migraine in Iran is also estimated at 14% (4). In comparison to the general population, a much higher prevalence of comorbid psychiatric disorders such as depression and anxiety has been reported in patients with migraine (5). Moreover, migraine negatively affects the quality of life and is known as one of the major causes of disability, mainly before the age of 50 years, throughout the world (6, 7). Given the pathophysiological complexity of migraine, limited progress has been made in disease management up to now (8). The importance of nitric oxide (NO) in the pathogenesis of migraine has been suggested (9). Moreover, the dilation of cerebral vessels and the inflammation of the trigeminovascular system inflammation, which occurs during migraine attacks, has been attributed to the role of NO in the pathogenesis of migraine (10). However, the exact pathophysiology of migraine is not well understood and therefore further attempts are needed to investigate pathogenesis-related determinants of migraine.

The potential association between diet and migraine has been recognized for many years (11, 12). Certain foods or their ingredients, such as alcohol, caffeine, dairy products, and citrus are known as common triggers of headache attacks in susceptible individuals (13, 14). Additionally, several observational studies have suggested an association between dietary intake of some nutrients such as folate, and magnesium with migraine (15–17). However, dietary patterns instead of single nutrients or foods will provide more comprehensive information regarding the association between diet and migraine (18). Recently, a limited number of observational studies have investigated the association between various dietary patterns and clinical characteristics of migraine. The results of a cross-sectional study showed that the frequency of migraine attacks is inversely associated with adherence to the healthy dietary pattern; while a positive correlation was found between adherence to the Western dietary pattern and the frequency of attacks (19). Additionally, previous studies have suggested that compliance with the dietary approaches to stop hypertension (DASH) diet and Mediterranean dietary patterns are associated with diminished symptoms of migraine headaches (20–22).

The limited number of evidence suggested a link between plant-based dietary patterns such as a low-fat vegan diet and the DASH diet and clinical characteristics of migraine (23, 24). However, further studies are necessary to comprehensively evaluate the potential association between plant-based eating patterns and migraine headaches without excluding all or several animal foods. In addition, it is important to note that the link between healthy plant foods and migraine may be different from those of less healthy plant foods. To address these issues, a plant-based diet index (PDI) that has been developed by Satija et al. (25) can be applied to comprehensively measure a different aspect of a plant-based diet of an individual. Hence, the present cross-sectional study was conducted among Iranian individuals diagnosed with migraine headaches to investigate the association between plant-based eating patterns and the clinical features of a migraine headache.

## Methods

### Study design and patients

The current cross-sectional study was carried out from August 2019 to June 2020 at Imam Musa Sadr and Khorshid Neurology Clinics, both affiliated with Isfahan University of Medical Sciences (IUMS), Isfahan, Iran. The study protocol was approved by the Research Ethics Committee of IUMS under the research number IR.MUI.RESEARCH.REC.1398.352. Before the participants’ enrollment, all patients received explanations of the study objectives and signed an informed consent form. The convenience sampling method was applied to select subjects. For this purpose, patients were evaluated against our eligibility criteria and those who met our criteria were enrolled in the current study. In total, 262 patients aged 20–50 years who met the International Classification of Headache Disorders 3 criteria were included in the study (26). The exclusion criteria were patients with a body mass index (BMI) lower than 18.5 or greater than 30 kg/m^2^; a history of diabetes, cancer, cardiovascular, thyroid, hepatic, renal, and neurological diseases; use of supplements such as feverfew, coenzyme Q10, magnesium, and riboflavin; and an estimated daily energy intake outside the normal range of 800 to 4,200 kcal/day based on the food frequency questionnaire (FFQ) report (27). Owing to the relationship between obesity and migraine (28), only non-obese migraine patients were recruited to increase the internal validity of the current study.

### Migraine-related outcomes

Migraine-related disability was assessed with a validated version of the headache impact test (HIT-6) questionnaire (29). This is a six-item questionnaire that asks questions regarding pain, social functioning, role functioning, vitality, cognitive functioning, and psychological functioning. Each item is scored on a five-point Likert scale (6 = never, 8 = seldom, 10 = occasionally, 11 = very often, and 13 = always). The final score of the questionnaire ranged from 36 to 78 with higher scores interpreted as a greater migraine-related disability (16). Additionally, patients were asked to fill out a 30-day headache diary, comprising information on the time of migraine attack onset, severity, and duration of headaches, during the upcoming month. The patients were asked to report headache severity based on a visual analog scale (VAS), where zero represented “no pain” and 10 represented “worst imaginable pain.” The number of attacks per month and the mean daily duration of attacks per month is defined as the frequency and duration of headache attacks.

### Dietary intake and calculation of PDI

Participants’ dietary intake was assessed using a validated 168-item, semi-quantitative FFQ (30). A trained dietitian, through a face-to-face interview, during the first visit asked patients to report their consumption frequency of each food item on a daily, weekly, or monthly basis during the previous year. Reported portion sizes of each food item in household measures converted to the gram per day (31, 32). Modified nutritionist IV software (First Databank, Hearst Corp, SanBruno, CA, United States) was used to analyze dietary food intakes.

Three versions of PDI comprising overall PDI, healthy PDI (hPDI), and unhealthy PDI (uPDI) were generated according to the previous report by Satija et al. (25). Generally, 18 food groups were created based on nutrient and culinary similarities and then categorized into three major groups of animal foods [meat (poultry and red meat), fish and seafood, egg, dairy foods, animal fats, and miscellaneous animal-based foods], healthy plant foods (whole grains, legumes, nuts, fruits, vegetables, vegetable oils, tea, and coffee), and less healthy plant foods (refined grains, potatoes, fruit juices, sugar-sweetened beverages, sweets, and desserts). Eighteen food groups were classified into quantiles and a score between 1 and 5 was assigned to each quantile. For PDIs, patients in the highest quantile of each food group received a score of 5 and those in the lowest quantile received a score of 1. Whereas in the animal food group, a score of 1 was assigned to patients in the highest quantile of consumption, while, a score of 5 was assigned to those in the lowest quantile. For hPDI, healthy plant food groups were given positive scores; while less healthy plant food and animal food groups were given reverse scores. For uPDI, less healthy plant food groups were given positive scores; while healthy plant food and animal food groups were given reverse scores. For every patient, scores of 18 food groups were summed to obtain the overall value.

### Other variables

A standard checklist was used to collect information regarding subjects’ age, gender, marital status, smoking status, family history of migraine, the number of family members, time since migraine diagnosis, migraine type (with/without aura and episodic/chronic), and medications. Episodic and chronic migraine are characterized by having headaches 0–14 and ≥ 15 days per month, respectively (33). The validated version of the international physical activity questionnaire (IPAQ) was used to estimate patients’ level of physical activity (34). The seven-item questionnaire provides information on the time spent on vigorous-intensity activity, moderate-intensity activity, walking, and sitting. Metabolic equivalents-minute per week (MET-min/week) was calculated by multiplying the total time spent on vigorous-intensity activity (minute), moderate-intensity activity, and walking by 8.0, 4.0, and 3.3 times, respectively. To calculate the overall physical activity score, three MET scores were summed. A trained dietitian measured anthropometric variables based on standard protocols. Weight was measured using an Omron BF511 digital scale (Omron Corp, Kyoto, Japan) while subjects wore light clothes and no shoes. An upstretched tape was used to measure height while subjects were in a standing position and wearing no shoes. BMI (kg/m^2^) was calculated as weight (kg) divided by squared height (m). Blood pressure (mmHg) was measured using a mercury sphygmomanometer (Riester, Germany) on the right arm in the sitting position in two stages with a 5-min interval. The average of two measurements was recorded as the final value. Mean arterial pressure (MAP) was estimated based on systolic and diastolic blood pressures using the following equation systolic blood pressure + 2 × diastolic blood pressure/3 (35).

Five milliliters of venous blood were taken from patients after 8–12 h of fasting. After the centrifuge at 3,500 rpm, the serum was separated and kept at −80°C for biochemical tests. Serum nitric oxide (NO) was measured using the Griess method with available commercial kits (Kiazist Life Sciences, Iran).

### Statistical analysis

The sample size was calculated based on the suggested formula for a cross-sectional design and similar studies via α = 0.05, β = 0.95, *r* = 0.25, and a drop-out rate of 10% that yielded 265 individuals (36). Data analysis was carried out using SPSS software version 21 (IBM Corp, Armonk, NY, United States). All the analysis was two-tailed and considered statistically significant if *p* values were lower than 0.05. The normal distribution of quantitative variables was assessed via the Q–Q plot, skewness test, and Kolmogorov–Smirnov test. Quantitative variables were expressed as mean ± standard error (SE) and categorical variables were presented as numbers (percentage). One-way ANOVA was implemented to examine the difference of quantitative variables across tertiles of PDI, hPDI, and uPDI. To assess the distribution of qualitative variables across tertiles of PDI, hPDI, and uPDI, the Chi-square test was used. The association between clinical features of migraine headaches and PDI, hPDI, and uPDI was investigated via multiple linear regression in different models, and beta (β) and 95% confidence interval (CI) were reported. The first model was adjusted for sex, age (continuous), and total energy intake (continuous). The second model additionally adjusted for marital status (single/married), smoking status (current smoker/non-current smoker), migraine characteristics (with aura/without aura), family history (yes/no), mean arterial pressure (continuous), medication (yes/no), and physical activity (continuous). Further adjustment was made for BMI in the third model.

## Results

A total of 262 participants (224 females and 38 males) enrolled in the current study with a mean (SE) age of 36.1 (0.53) years, a BMI of 25.55 (0.21) kg/m^2^, and a total energy intake of 2651.99 kcal/day. General characteristics of the study population across tertiles of PDI, hPDI, and uPDI are shown in Table 1. Patients in the highest tertile of PDI compared to the lowest tertile had a higher headache frequency (*p* = 0.010) and a lower serum NO level (*p* = 0.035). Moreover, they were more likely to have a family history of migraine (*p* = 0.030) and take tricyclic antidepressants (*p* = 0.025). Individuals in the first tertile of hPDI compared to the third tertile were more likely to be younger (*p* < 0.001), male (p < 0.001), single (*p* = 0.017), current smoker (*p* = 0.014), and beta-blocker user (*p* = 0.027). Patients in the highest tertile of hPDI had a higher time since migraine diagnosis (*p* = 0.002) and lower serum NO levels (*p* = 0.033) and height (*p* = 0.003). Moreover, they were more likely to take triptans (*p* = 0.010). Participants in the highest tertile of uPDI compared to the lowest tertile had a lower weight (*p* = 0.002) and height (*p* = 0.002). No other significant difference was observed for any of the studied parameters across tertiles of PDI, hPDI, and uPDI.

**Table 1:** Characteristics of the study population stratified by tertiles of plant-based diet index (PDI), healthy plant-based diet index (hPDI), and unhealthy plant-based diet index (uPDI).

Variables	PDI				hPDI				uPDI			
	T1	T2	T3	P value	T1	T2	T3	P value	T1	T2	T3	P value
N	90	82	90		90	91	81		88	92	82	
*Demographic variables*												
Age (y)	35.20 ± 0.99	37.21 ± 0.93	35.98 ± 0.82	0.306	32.85 ± 0.94	36.63 ± 0.85	39.11 ± 0.83	<0.001	34.96 ± 0.78	36.01 ± 0.95	37.42 ± 1.00	0.177
Female	72 (80.0)	71 (86.6)	81 (90.0)	0.057	63 (70.0)	85 (93.4)	76 (93.8)	<0.001	72 (81.8)	78 (84.8)	74 (90.2)	0.121
Married	70 (77.8)	67 (81.7)	75 (83.3)	0.344	64 (71.1)	79 (86.8)	69 (85.2)	0.017	73 (83.0)	72 (78.3)	67 (81.7)	0.823
Current smoker	4 (4.4)	7 (8.5)	4 (4.4)	>0.99	10 (11.1)	3 (3.3)	2 (2.5)	0.014	5 (5.7)	6 (6.5)	4 (4.9)	0.829
Number of family members	3.36 ± 0.10	3.42 ± 0.09	3.45 ± 0.11	0.829	3.43 ± 0.10	3.35 ± 0.10	3.46 ± 0.10	0.726	3.28 ± 0.10	3.39 ± 0.09	3.58 ± 0.11	0.134
Weight (kg)	68.90 ± 1.14	66.74 ± 1.25	67.72 ± 1.05	0.417	68.88 ± 1.32	66.17 ± 0.95	68.49 ± 1.12	0.190	71.01 ± 1.06	66.84 ± 1.16	65.49 ± 1.13	0.002
Height (cm)	164.17 ± 0.86	161.54 ± 0.85	162.72 ± 0.77	0.086	165.10 ± 0.94	161.76 ± 0.61	161.58 ± 0.87	0.003	164.84 ± 0.80	162.89 ± 0.81	160.67 ± 0.83	0.002
BMI (kg/m^2^)	25.52 ± 0.33	25.56 ± 0.42	25.56 ± 0.34	0.996	25.20 ± 0.38	25.30 ± 0.35	26.20 ± 0.35	0.117	26.10 ± 0.33	25.19 ± 0.38	25.36 ± 0.37	0.170
Physical activity (MET/h/d)	7.91 ± 1.83	10.92 ± 3.19	7.98 ± 1.44	0.562	12.93 ± 3.25	6.68 ± 1.39	6.84 ± 1.19	0.072	8.61 ± 1.54	10.60 ± 2.88	7.22 ± 1.89	0.558
SBP (mmHg)	113.46 ± 0.92	113.71 ± 0.89	110.88 ± 1.23	0.101	111.88 ± 1.04	113.73 ± 0.84	112.30 ± 1.23	0.411	112.04 ± 1.14	112.52 ± 1.04	113.47 ± 0.90	0.625
DBP (mmHg)	75.50 ± 0.81	75.75 ± 0.78	75.22 ± 0.72	0.891	74.42 ± 0.82	76.58 ± 0.66	75.43 ± 0.83	0.134	75.30 ± 0.76	75.05 ± 0.80	76.15 ± 0.75	0.584
MAP (mmHg)	88.15 ± 0.78	88.41 ± 0.74	87.11 ± 0.82	0.467	86.91 ± 0.80	88.96 ± 0.67	87.72 ± 0.88	0.168	87.55 ± 0.81	87.54 ± 0.80	88.59 ± 0.73	0.567
*Migraine-related information*												
Migraine in first-degree relatives	49 (54.4)	55 (67.1)	63 (70.0)	0.030	57 (63.3)	60 (65.9)	50 (61.7)	0.841	63 (71.6)	56 (60.9)	48 (58.5)	0.075
Time since migraine diagnosis (y)	6.42 ± 0.90	7.22 ± 0.88	8.34 ± 0.99	0.331	4.89 ± 0.84	7.76 ± 0.91	9.56 ± 0.98	0.002	6.98 ± 0.79	7.00 ± 0.96	8.08 ± 1.02	0.643
Episodic migraine	69 (76.7)	73 (89.0)	74 (82.2)	0.328	70 (77.8)	76 (83.5)	70 (86.4)	0.136	74 (84.1)	75 (81.5)	67 (81.7)	0.679
Migraine with aura	31 (34.4)	35 (42.7)	43 (47.8)	0.070	39 (43.3)	36 (39.6)	34 (42.0)	0.846	41 (46.6)	36 (39.1)	32 (39.0)	0.312
Frequency (attacks per month)	9.57 ± 0.90	7.17 ± 0.74	6.61 ± 0.50	0.010	8.70 ± 0.76	6.92 ± 0.71	7.80 ± 0.76	0.234	7.86 ± 0.70	8.03 ± 0.73	7.48 ± 0.82	0.874
Duration (day/attack)	0.92 ± 0.08	0.93 ± 0.09	1.03 ± 0.09	0.665	0.90 ± 0.08	1.00 ± 0.09	0.98 ± 0.08	0.672	0.95 ± 0.08	1.01 ± 0.08	0.92 ± 0.08	0.782
Severity (visual analog scale)	7.68 ± 0.18	7.81 ± 0.19	7.83 ± 0.19	0.840	7.58 ± 0.19	7.74 ± 0.18	8.02 ± 0.18	0.275	7.90 ± 0.19	7.63 ± 0.16	7.80 ± 0.21	0.571
HIT-6	63.10 ± 0.76	62.50 ± 0.81	62.54 ± 0.73	0.827	62.28 ± 0.79	63.52 ± 0.70	62.29 ± 0.80	0.416	63.37 ± 0.71	62.02 ± 0.78	62.80 ± 0.80	0.447
Nitric oxide (nmol/mL)	36.69 ± 2.36	36.48 ± 2.57	29.42 ± 1.82	0.035	36.55 ± 2.24	36.30 ± 2.53	29.00 ± 1.86	0.033	30.89 ± 2.04	35.51 ± 2.28	36.05 ± 2.49	0.214
*Medications0*												
Taking beta-blockers	38 (42.2)	36 (43.9)	34 (37.8)	0.545	50 (55.6)	26 (28.6)	32 (39.5)	0.027	36 (40.9)	41 (44.6)	31 (37.8)	0.694
Taking topiramate	6 (6.7)	3 (3.7)	4 (4.4)	0.493	5 (5.6)	4 (4.4)	4 (4.9)	0.846	5 (5.7)	6 (6.5)	2 (2.4)	0.341
Taking TCAs	35 (38.9)	37 (45.1)	50 (55.6)	0.025	44 (48.9)	39 (42.9)	39 (48.1)	0.901	39 (44.3)	47 (51.1)	36 (43.9)	0.975
Taking TeCAs	2 (2.2)	3 (3.7)	3 (3.3)	0.665	2 (2.2)	3 (3.3)	3 (3.7)	0.571	3 (3.4)	3 (3.3)	2 (2.4)	0.716
Taking SNRIs	7 (7.8)	4 (4.9)	3 (3.3)	0.186	7 (7.8)	4 (4.4)	3 (3.7)	0.232	6 (6.8)	7 (7.6)	1 (1.2)	0.111
Taking sodium valproate	11 (12.2)	10 (12.2)	12 (13.3)	0.823	13 (14.4)	13 (14.3)	7 (8.6)	0.263	11 (12.5)	9 (9.8)	13 (15.9)	0.525
Taking triptans	17 (18.9)	12 (14.6)	14 (15.6)	0.547	8 (8.9)	16 (17.6)	19 (23.5)	0.010	14 (15.9)	13 (14.1)	16 (19.5)	0.537
Taking gabapentin	15 (16.7)	14 (17.1)	14 (15.6)	0.841	11 (12.2)	14 (15.4)	18 (22.2)	0.080	16 (18.2)	12 (13.0)	15 (18.3)	0.997
Taking benzodiazepine	5 (5.6)	5 (6.1)	3 (3.3)	0.493	6 (6.7)	3 (3.3)	4 (4.9)	0.585	2 (2.3)	5 (5.4)	6 (7.3)	0.130

Data are presented as mean±standard error or number (% within tertiles of dietary PDI, hPDI, uPDI)

The P-value was obtained from chi-square analysis for categorical variables and analysis of variance (ANOVA) for continuous variables.

BMI: Body mass index, SBP: Systolic Blood Pressure; DBP: Diastolic Blood Pressure; MAP: Mean Arterial Pressure; TCA: Tricyclic Antidepressants; TeCA: Tetracyclic Antidepressant; SNRI: Serotonin-Norepinephrine Reuptake Inhibitor, HIT: Headache Impact Test, PDI: Plant-based diet index, hPDI: Healthy plant-based diet index, uPDI: Unhealthy plant-based diet index

Selected food groups and nutrient intake of participants across tertiles of PDI, hPDI, and uPDI are presented in Table 2. Patients in the highest tertile of PDI consumed higher amounts of carbohydrate (*p* < 0.001), total fiber (*p* < 0.001), potassium (*p* = 0.020), and magnesium (*p* = 0.004). Participants with the highest score of hPDI consumed lower amounts of total energy (*p* < 0.001), protein (*p* < 0.001), fat (*p* < 0.001), carbohydrate (*p* < 0.001), and calcium (*p* = 0.007). Moreover, patients in the highest tertile of hPDI consumed higher amounts of magnesium (*p* = 0.031). Subjects in the highest tertile of uPDI consumed lower amounts of total energy (*p* < 0.001), carbohydrate (*p* = 0.001), fat (*p* < 0.001), protein (*p* < 0.001), total fiber (*p* < 0.001), sodium (*p* = 0.023), potassium (*p* < 0.001), calcium (*p* < 0.001), and magnesium (*p* < 0.001).

**Table 2:** Selected food groups and nutrient intake of participants across tertiles of plant-based diet index (PDI), healthy plant-based diet index (hPDI), and unhealthy plant-based diet index (uPDI).

Variables	PDI				hPDI				uPDI			
	T1	T2	T3	P-value	T1	T2	T3	P-value	T1	T2	T3	P-value
Mean score	48.22 ± 0.38	55.08 ± 0.14	61.67 ± 0.36	<0.001	47.96 ± 0.43	57.03 ± 0.18	65.25 ± 0.43	<0.001	46.65 ± 0.46	56.04 ± 0.21	64.19 ± 0.37	<0.001
*Nutrients*												
Energy (kcal/day)	2546.32 ± 69.54	2654.26 ± 76.13	2756.86 ± 68.55	0.113	2990.13 ± 71.36	2589.79 ± 56.38	2374.37 ± 70.42	<0.001	2941.23 ± 71.43	2663.55 ± 67.18	2358.46 ± 60.90	<0.001
Protein (g/d)	74.01 ± 3.63	71.54 ± 3.17	74.11 ± 2.68	0.815	84.97 ± 3.30	71.20 ± 2.83	63.48 ± 2.94	<0.001	87.52 ± 3.54	75.12 ± 2.69	57.37 ± 2.22	<0.001
Fat (g/d)	113.43 ± 3.33	111.88 ± 3.76	107.36 ± 3.76	0.404	123.52 ± 3.28	108.48 ± 2.92	100.63 ± 3.23	<0.001	123.95 ± 3.05	112.88 ± 3.21	96.10 ± 2.85	<0.001
Carbohydrate (g/d)	325.52 ± 10.42	361.04 ± 11.77	398.16 ± 11.79	<0.001	406.37 ± 11.54	352.52 ± 10.21	325.21 ± 11.96	<0.001	395.27 ± 12.43	358.32 ± 10.32	331.76 ± 11.71	0.001
Total fiber (g/d)	16.55 ± 0.65	19.90 ± 0.84	23.02 ± 0.81	<0.001	18.94 ± 0.78	19.14 ± 0.70	21.46 ± 0.98	0.061	24.89 ± 0.92	18.82 ± 0.59	15.91 ± 0.58	<0.001
Sodium (mg/d)	6737.05 ± 176.42	7012.10 ± 158.27	6936.47 ± 183.07	0.514	7136.40 ± 190.50	6773.39 ± 167.04	6752.51 ± 157.25	0.210	6943.34 ± 198.03	7180.05 ± 131.43	6512.57 ± 183.29	0.023
Potassium (mg/d)	3439.82 ± 125.02	3613.73 ± 138.43	3951.49 ± 133.27	0.020	3821.87 ± 135.45	3583.40 ± 121.25	3598.59 ± 145.06	0.363	4365.83 ± 138.91	3587.07 ± 101.36	3016.35 ± 119.51	<0.001
Calcium (mg/d)	1068.29 ± 64.12	1027.61 ± 55.15	980.42 ± 50.92	0.550	1166.90 ± 57.18	990.17 ± 53.73	920.29 ± 57.27	0.007	1178.82 ± 61.04	1037.33 ± 51.80	864.25 ± 53.17	<0.001
Magnesium (mg/d)	254.34 ± 9.95	279.43 ± 10.35	301.71 ± 9.98	0.004	268.22 ± 11.26	266.90 ± 8.94	300.85 ± 10.46	0.031	330.98 ± 11.10	276.93 ± 8.01	228.73 ± 8.21	<0.001

Data are presented as mean ± standard error and obtained from analysis of variance (ANOVA).

P<0.05 was considered statistically significant.

PDI: Plant-based diet index, hPDI: Healthy plant-based diet index, uPDI: Unhealthy plant-based diet index

Beta (β) and 95% CI for clinical features of migraine headaches across tertiles of PDI, hPDI, and uPDI are given in Table 3. In the crude model, PDI was negatively associated with a lower headache frequency for patients in the third tertile of PDI compared to those in the first tertile (β = −2.96; 95% CI: −4.97, −0.96; *p*_trend_ = 0.004). This association remained also significant after adjustment for sex, age, total energy intake, marital status, smoking status, migraine characteristic, family history, MAP, medication, and physical activity (β = −2.98; 95% CI: −5.00, −0.96; *p*_trend_ = 0.004). Following further adjustment for BMI, an increase in mean PDI score from 48.22 to 61.67 decreased the frequency of headaches by −2.95 (95% CI: 0.06, 0.53; *p*_trend_ = 0.005). PDI was inversely associated with serum levels of NO in those with the highest scores of PDI compared to those with the lowest scores (β = −7.26; 95% CI: −13.39, −1.13; *p*_trend_ = 0.021). This association was also observed after further adjustment for sex, age, total energy intake, marital status, smoking status, migraine characteristics, family history, mean arterial pressure, medication, physical activity, and BMI (β = −7.69; 95% CI: −14.68, −0.69; *p*_trend_ = 0.028). No significant association was observed between PDI and headaches duration either before (β = 0.10; 95% CI: −0.14, 0.34; *p*_trend_ = 0.415) or after adjustment for potential confounders (β = 0.05; 95% CI: −0.21, 0.32; *p*_trend_ = 0.668) in people with the highest score of PDI compared to those with the lowest values. Moreover, no significant relationship was detected between PDI and migraine-related disability and headaches severity.

**Table 3:** Beta (β) and 95% confidence interval for clinical features of migraine headache according to tertiles of plant-based diet index (PDI), healthy plant-based diet index (hPDI), and unhealthy plant-based diet index (uPDI).

	PDI				hPDI				uPDI			
	T1	T2	T3	P trend	T1	T2	T3	P trend	T1	T2	T3	P trend
Frequency Crude Model 1 Model 2 Model 3	Ref Ref Ref Ref	-2.40 (-4.46, -0.35) -1.82 (-3.97, 0.31) -2.05 (-4.10, -0.01) -2.01 (-4.07, 0.03)	-2.96 (-4.97, -0.96) -2.56 (-4.69, -0.44) -2.98 (-5.00, -0.96) -2.95 (-4.97, -0.92)	0.004 0.019 0.004 0.005	Ref Ref Ref Ref	-1.77 (-3.80, 0.24) -0.20 (-2.46, 2.07) 0.50 (-1.63, 2.64) 0.51 (-1.63, 2.65)	-0.89 (-2.98, 1.18) 0.59 (-1.85, 3.04) 0.52 (-1.76, 2.82) 0.52 (-1.77, 2.82)	0.376 0.615 0.662 0.666	Ref Ref Ref Ref	0.16 (-1.87, 2.21) 0.69 (-1.47, 2.86) 0.39 (-1.78, 2.56) 0.41 (-1.76, 2.59)	-0.37 (-2.47, 1.72) 0.21 (-2.12, 2.54) -0.19 (-2.53, 2.14) -0.17 (-2.52, 2.16)	0.732 0.862 0.864 0.873
Duration Crude Model 1 Model 2 Model 3	Ref Ref Ref Ref	0.006 (-0.24, 0.25) 0.002 (-0.27, 0.27) 0.004 (-0.27, 0.27) -0.008 (-0.28, 0.26)	0.10 (-0.14, 0.34) 0.05 (-0.21, 0.32) 0.06 (-0.20, 0.33) 0.05 (-0.21, 0.32)	0.415 0.687 0.623 0.668	Ref Ref Ref Ref	0.10 (-0.13, 0.34) -0.09 (-0.38, 0.19) -0.13 (-0.41, 0.15) -0.13 (-0.41, 0.14)	0.08 (-0.16, 0.33) -0.11 (-0.42, 0.19) -0.10 (-0.40, 0.20) -0.09 (-0.39, 0.20)	0.506 0.495 0.541 0.561	Ref Ref Ref Ref	0.05 (-0.18, 0.29) 0.02 (-0.25, 0.29) 0.06 (-0.21, 0.33) 0.05 (-0.22, 0.32)	-0.03 (-0.28, 0.21) -0.05 (-0.35, 0.23) 0.005 (-0.29, 0.30) 0.001 (-0.29, 0.29)	0.811 0.709 0.981 0.995
Severity Crude Model 1 Model 2 Model 3	Ref Ref Ref Ref	0.12 (-0.40, 0.66) 0.10 (-0.45, 0.66) 0.16 (-0.39, 0.73) 0.18 (-0.38, 0.74)	0.14 (-0.37, 0.66) -0.12 (-0.68, 0.42) -0.07 (-0.63, 0.48) -0.06 (-0.62, 0.49)	0.586 0.639 0.767 0.793	Ref Ref Ref Ref	0.15 (-0.35, 0.67) -0.08 (-0.67, 0.49) -0.22 (-0.80, 0.36) -0.21 (-0.79, 0.37)	0.43 (-0.09, 0.96) 0.21 (-0.41, 0.84) 0.09 (-0.52, 0.72) 0.08 (-0.53, 0.70)	0.110 0.474 0.709 0.749	Ref Ref Ref Ref	-0.27 (-0.79, 0.24) -0.17 (-0.73, 0.38) -0.08 (-0.64, 0.47) -0.07 (-0.63, 0.48)	-0.10 (-0.63, 0.43) -0.03 (-0.63, 0.56) 0.02 (-0.58, 0.62) 0.02 (-0.57, 0.63)	0.687 0.915 0.939 0.924
HIT-6 Crude Model 1 Model 2 Model 3	Ref Ref Ref Ref	-0.60 (-2.74, 1.54) -0.11 (-2.44, 2.21) -0.25 (-2.61, 2.11) -0.45 (-2.82, 1.90)	-0.55 (-2.64, 1.53) -0.74 (-3.05, 1.56) -0.99 (-3.33, 1.34) -1.16 (-3.48, 1.16)	0.603 0.522 0.399 0.326	Ref Ref Ref Ref	1.23 (-0.84, 3.31) 1.41 (-1.01, 3.84) 1.52 (-0.85, 3.90) 1.45 (-0.92, 3.82)	0.007 (-2.13, 2.15) 0.31 (-2.30, 2.94) -0.31 (-2.86, 2.23) -0.21 (-2.76, 2.33)	0.962 0.863 0.737 0.809	Ref Ref Ref Ref	-1.35 (-3.44, 0.73) -0.61 (-2.95, 1.71) -0.38 (-2.74, 1.98) -0.50 (-2.85, 1.84)	-0.57 (-2.71, 1.57) -0.12 (-2.62, 2.37) 0.08 (-2.45, 2.62) -0.01 (-2.54, 2.51)	0.587 0.924 0.944 0.999
Nitric oxide Crude Model 1 Model 2 Model 3	Ref Ref Ref Ref	-0.20 (-6.48, 6.06) 0.45 (-6.48, 7.40) 0.26 (-6.81, 7.33) -0.05 (-7.14, 7.04)	-7.26 (-13.39, -1.13) -7.13 (-14.01, -0.25) -7.44 (-14.42, -0.45) -7.69 (-14.68, -0.69)	0.021 0.040 0.033 0.028	Ref Ref Ref Ref	-0.24 (-6.35, 5.86) -3.95 (-11.15, 3.24) -4.29 (-11.59, 3.00) -4.29 (-11.59, 3.00)	-7.55 (-13.84, -1.25) -12.06 (-19.83, -4.29) -11.92 (-19.74, -4.10) -11.92 (-19.74, -4.10)	0.021 0.002 0.003 0.003	Ref Ref Ref Ref	4.62 (-1.55, 10.79) 3.77 (-3.23, 10.79) 3.90 (-3.21, 11.02) 3.74 (-3.37, 10.87)	5.15 (-1.19, 11.51) 4.42 (-3.11, 11.96) 4.78 (-2.87, 12.44) 4.66 (-2.99, 12.32)	0.109 0.251 0.225 0.237

Data are presented as β (95% confidence interval) and obtained from linear regression.

P<0.05 was considered statistically significant.

Crude: Unadjusted

Model 1: Adjusted for age, sex, and energy intake

Model 2: Model 1+ marital status, smoking status, migraine characteristic, family history, mean arterial pressure, medication, and physical activity.

Model 3: Model 2 + body mass index

HIT: Headache Impact Test, PDI: Plant-based diet index, hPDI: Healthy plant-based diet index, uPDI: Unhealthy plant-based diet index

In the crude model, hPDI was associated with a lower serum NO in those with the highest adherence to a healthy plant-based dietary pattern compared to those with the lowest adherence (β = −7.55; 95% CI: −13.84, −1.25; *p*_trend_ = 0.021). After adjustment for potential confounders, an increase in the mean hPDI score from 47.96 to 65.25 decreased serum levels of NO by −11.92 (95% CI: −19.74, −4.10; *p*_trend_ = 0.003). No significant association was observed between hPDI and scores of HIT-6 and clinical outcomes of migraine including frequency, duration, and severity.

After controlling for potential confounders, no significant relationship was detected between the score of uPDI and studied outcomes including headaches frequency (β = −0.17; 95% CI: −2.52, 2.16; *p*_trend_ = 0.873), duration (β = 0.001; 95% CI: −0.29, 0.29; *p*_trend_ = 0.995), severity (β = 0.02; 95% CI: −0.57, 0.63; *p*_trend_ = 0.924), migraine-related disability (β = −0.01; 95% CI: −2.54, 2.51; *p*_trend_ = 0.999), and serum levels of NO (β = 4.66; 95% CI: −2.99, 12.32; *p*_trend_ = 0.237).

## Discussion

Traditionally, foods were categorized into plant and animal sources, and plant-based foods were generally believed to be a substantial part of a healthy lifestyle (37). However, further research proposed that not all types of plant foods should be considered healthy; refined grains, sugar-sweetened beverages, and potatoes are plant-based foods with undesirable effects in terms of diabetes and cardiovascular disease (38–42). Therefore, it was hypothesized that the type of plant foods also might be important concerning migraine headaches, and subsequently, a plant-based dietary pattern was examined using PDI, hPDI, and uPDI to have a better insight into this issue.

The current study was carried out to investigate the possible role of a plant-based eating style in migraine patients in terms of headache frequency, duration, severity, migraine-related disability, and serum levels of NO. It was found that there is a negative association between PDI and headache frequency and serum NO levels. Moreover, an inverse relationship was detected between hPDI and serum NO levels. No other significant association was observed between any of the studied parameters and PDI, hPDI, and uPDI. Our findings, although preliminary, suggest the importance of dietary patterns in the pathophysiology and management of migraine headaches and may contribute to the existing literature and provide new and substantial information regarding the association between plant-based dietary patterns and migraine headaches in a sample of the Iranian population. Moreover, our findings proposed that the association between plant-based dietary patterns and migraine outcomes was independent of studied confounders including sex, age, total energy intake, marital status, smoking status, migraine characteristic, family history, and mean arterial pressure, medication, physical activity, and BMI.

A case of a 60-year-old man with a confirmed diagnosis of chronic headaches provided a shred of promising evidence for following a plant-based diet in terms of headache frequency (43). A cross-sectional survey of 266 Iranian women (18–50 years) also provided a significant association between hPDI and headache severity, duration, and migraine-related disability; however, it failed to support our findings in terms of headache frequency (44). Another cross-over trial among 42 migraine sufferers also suggested a beneficial effect of a vegan diet combined with an elimination diet during a 36-week intervention in terms of headache severity and duration (23). Moreover, a DASH diet was shown to be efficacious in terms of frequency and severity among 102 women with migraine (45). As can be conjectured from the available literature, there is limited evidence in terms of PDI and migraine; however, reviewed studies imply the beneficial role of a plant-based eating style.

Some mechanisms may justify the observed association between plant-based dietary patterns and clinical outcomes of migraine headaches. Plant-based diets are generally low in sodium and have a lower sodium-to-potassium ratio unless it was added during food preparation (46). A substantial role of sodium in migraine pathophysiology was suggested by previous investigations (47–50). Sodium is increased during attacks in the blood (51) and cerebrospinal fluid (CSF) of migraine patients (52). Increased extracellular sodium levels can inactivate voltage-gated sodium channels subsequently leading to a reduction of resting membrane potential and therefore threshold for action potentials (53). Sulforaphane and glucosinolate which are the natural components of some vegetables, such as carrots, spinach, parsley, beets, broccoli, and cabbage were found to be effective in antagonizing calcitonin gene-related peptide (CGRP) (54, 55). CGRP can cause neurogenic inflammation, which is found during a migraine attack, and also act as a strong vasodilator (56, 57). It was proposed that neuroinflammation accompanied by sensitization of nerve endings may play a substantial role in the pathophysiology of migraine (58). A plant-based diet is loaded with phytochemicals, antioxidants, and dietary fibers with anti-inflammatory properties (59). Dietary fibers reduce inflammation by suppressing the production of inflammatory cytokines, altering gut microflora, and slowing glucose absorption (27). A plant-based eating style is generally accompanied by higher consumption of magnesium as it can be found in whole grains, seeds, nuts, legumes, and green leafy vegetables, such as spinach (60). Previous documents reported that migraine patients may have magnesium deficiency and also low brain magnesium during attacks (61, 62). In addition, previous studies proposed the reduction of prostacyclin-dependent vasodilation, blocking of N-Methyl-D-aspartate (NMDA) receptors, and inhibition of serotonin-dependent vascular spasm as possible mechanisms of magnesium in the context of migraine prevention (63).

Nitric oxide disappear within seconds due to its reaction with free oxygen. Therefore, measuring its metabolites with a longer half-life is essential in future studies. The half-lives of nitrate and nitrite are 5–8 h and 20–45 min, respectively (64). However, dietary nitrate and nitrite intakes should be controlled in future investigations (65).

The current study is among the first investigations that examined the association between PDI and migraine outcomes in a sample of the Iranian population; however, some limitations warrant consideration. It was a cross-sectional survey that precludes us to investigate a cause-and-effect association between plant-based eating styles and migraine. Our findings proposed that the observed association was independent of potential confounders including sex, age, total energy intake, marital status, smoking status, migraine characteristics, family history, mean arterial pressure, medication, physical activity, and BMI; however, the effects of residual confounders cannot be excluded. Therefore, further studies on controlling additional confounders are needed. Although we used a validated version of a 168-item FFQ, the possibility of misclassification might affect our findings. In addition, FFQ is prone to recall bias. Moreover, we were unable to assess response bias, due to a lack of data on non-responders.

## Conclusion

Our findings suggest that higher adherence to a plant-based eating style, as evidenced by higher PDI and hPDI values, was significantly associated with lower headache frequency and serum levels of NO. Further studies are needed to confirm our findings and also to provide a causative relationship between plant-based dietary patterns and migraine outcomes.

## Data availability statement

The raw data supporting the conclusions of this article will be made available by the authors, without undue reservation.

## Ethics statement

The study protocol was approved by the Research Ethics Committee of IUMS under the research number of IR.MUI.RESEARCH.REC.1398.352. The patients/participants provided their written informed consent to participate in this study.

## Author contributions

ArA, GA, and EK: conception and design. ArA, EK, and ST: acquisition of the data. ArA: analysis and interpretation of the data and revising it for intellectual content. ArA, EK, ST, and EA: drafting the manuscript. All authors contributed to the article and approved the submitted version.

## Funding

This study was funded by the Isfahan University of Medical Sciences, Isfahan, Iran.

## Conflict of interest

The authors declare that the research was conducted in the absence of any commercial or financial relationships that could be construed as a potential conflict of interest.

## Publisher’s note

All claims expressed in this article are solely those of the authors and do not necessarily represent those of their affiliated organizations, or those of the publisher, the editors and the reviewers. Any product that may be evaluated in this article, or claim that may be made by its manufacturer, is not guaranteed or endorsed by the publisher.
